# Shear Capacity of Hollow High-Performance Concrete Beams with Cross-Wound Carbon Fiber-Reinforced Polymer Reinforcement

**DOI:** 10.3390/polym17010075

**Published:** 2024-12-30

**Authors:** Tomáš Vlach, Jakub Řepka, Jakub Hájek, Jan Pošta, Richard Fürst, Petr Hájek

**Affiliations:** 1 University Centre for Energy Efficient Buildings, Czech Technical University in Prague, 27343 Buštěhrad, Czech Republic; jakub.repka@cvut.cz (J.Ř.); jakub.hajek@cvut.cz (J.H.); jan.posta@cvut.cz (J.P.); petr.hajek@fsv.cvut.cz (P.H.); 2 Federal Institute for Materials Research and Testing (BAM), Division 7.3-Fire Engineering, Unter den Eichen 87, 12205 Berlin, Germany; richard.fuerst@bam.de

**Keywords:** hollow concrete beam, shear reinforcement, composite reinforcement, woven reinforcement, cross-wound reinforcement, fiber-reinforced polymer, high-performance concrete

## Abstract

This paper introduces cross-wound CFRP shear reinforcement of hollow HPC beams. The CFRP reinforcement was manufactured in the form of a square tubular mesh from carbon rovings oriented at ±45° from the longitudinal axis. The shear reinforcement was made in two variants from carbon yarns with linear densities of 1600 and 3700 tex. Tensile reinforcement made of BFRP bars was positioned directly around the hollow core and was used as a platform for manual winding of the shear reinforcement. The hollow beams were subjected to a three-point bending test with four configurations of the tensile BFRP reinforcement for better evaluation of the effect of the shear reinforcement under different conditions. The 1600 tex shear reinforcement increased the ultimate flexural strength by at least 89% compared to specimens without any shear reinforcement. The 3700 tex shear reinforcement yielded slightly better results in most cases but was not utilized to its full shear capacity as these specimens always failed in shear due to the delamination of the concrete matrix from the shear reinforcement. There was too much reinforcement in the beam cross-section.

## 1. Introduction

In view of the increasing number of natural and man-made disasters and the increasing economic and social problems, it is necessary to adapt the existing principles and methods of structural design, the corresponding construction techniques, and the operation of buildings to make them more sustainable, resilient, and adaptable to new situations in changing natural and socio-economic conditions in the world. Concrete, in general, is the most used material in construction. Recent research and development of concrete composition, production technology, and development of concrete constructions, intensified over the last 20 years, have led to the improvement of technical parameters while reducing environmental impacts. Due to the optimization of the mixture, new types of concrete can have significantly better characteristics from the perspective of strength, mechanical resistance, durability, and resistance to extreme loads. However, it is necessary to further search for the effective application of these new silicate materials in wide practice. Developed technical solutions could contribute to addressing the Sustainable Development Goals (SDGs), which the United Nations set out in 2015 as a 2030 action plan [1].

Concrete is gradually becoming a building material with a high potential for technical solutions meeting these new requirements, leading to the necessary reduction in environmental impacts and the consequent necessary improvement of properties in the conditions of a changing climate. With respect to the amount of produced concrete, the optimization of concrete structures represents a great potential for improving the complex quality of structures from the perspective of sustainable development [2,3,4,5]. One of the ways can be a completely new look at concrete structures and the search for the optimal application of new silicate materials or their combinations. It can be a sandwich construction, combinations of concrete, combinations of reinforcements, or shape optimization using the potential of new silicate materials and alternative reinforcement.

This article presents an example of the last-mentioned variant. It uses the advantages of high-performance concrete (HPC) with its high mechanical performance and durability [6,7,8] in combination with alternative composite reinforcement in the form of hollow beams with hybrid basal fiber-reinforced polymer (BFRP) bars and carbon fiber-reinforced polymer (CFRP) woven reinforcement. This non-corrosive reinforcement also has high mechanical performance and higher durability [9,10] in comparison with traditional steel reinforcement. Thanks to that, elements can be designed only for load-bearing capacity, not durability, leading to extremely thin elements without considering traditional reinforcement concrete cover thicknesses. To maximize this effect and minimize material consumption, it is appropriate to optimize the shape of such cross-sections, for example, the I-shape [11,12], I-shape as a structural element [13,14,15]. Another option for lightening and optimizing is the use of a hollow cross-section, which corresponds to the presented topic. It is possible to use a hollow section in both columns and beams, very often with a tubular shape [16,17,18,19].

Flexural behavior is relatively well described and known; it is interesting to focus more closely on the shear capacity of thin-walled hollow HPC beams with BFRP reinforcement bars and CFRP woven reinforcement. The application technology, the way of shaping the shear reinforcement, and its angle compared to traditional solutions are original. Textile concrete reinforcement of both commercial and laboratory production is capable of reliably transmitting shear stress, as presented in the mentioned articles [11,12,13,14,15,16,17,18,19]. All the above solutions use the longitudinal and transverse directions of individual textile reinforcement roving, i.e., angles of 0 and 90 degrees from the longitudinal direction of the 1D element, not 45 degrees, as presented in the presented article. There are also articles generally describing FRP shear reinforcement in the form of stirrups [20,21] or spirals [22,23,24] with open or closed-type winding, which is a conservative approach for massive structures close to the traditional approach to metal reinforcement from the point of view of static action, which is not very suitable for hollow ultra-thin HPC elements. Traditional composite reinforcement allows for the minimization of the concrete cover layer in terms of durability. However, to ensure sufficient interaction with the cementitious matrix, a greater thickness of the concrete layer is required. The cross-wound consists of individual roving, making it suitable for extremely thin elements with a concrete layer thickness of only about one centimeter. A separate category is additional reinforcement of elements using FRP and textiles; however, it does not correspond to the production technology of the presented beams [25,26,27] because the article presents newly created elements.

The paper focuses on homogenized yarn to maximize the efficiency of carbon fibrils using epoxy resin [28,29,30] also with reinforcement surface treatment [28,30,31,32,33]. The polymer matrix of yarn can also protect single fibrils against the alkaline environment of concrete [34,35,36], but also has some negatives like lover resistance [37] to elevated temperature. It is also possible to replace the resin using cementitious [38] or geopolymer suspension for the yarn homogenization [39].

Thanks to the method of execution and the angle of 45 degrees of the reinforcement from the longitudinal direction, this reinforcement not only resists shear loading but also participates in the transmission of tensile stress at the bottom surface. The carbon yarn is applied around BFRP bending reinforcement bars on the removable rigid steel core, which forms the shape of the cross-section. The carbon yarn forms a square mesh oriented at 45° to the element axis. The density of the mesh corresponds to the number of yarns in the cross-section. The mesh is then homogenized using epoxy resin; this step connects the carbon mesh and the BFRP bars into one solid whole. The surface of CFRP mesh is coated using fine-grain silica sand.

The samples have a shear reinforcement of two types, which differ only in the linear density of used carbon yarn. The spacing of the woven reinforcement and the number of rovings are identical for all specimens. Each group was supplemented with samples without any woven reinforcement to clearly demonstrate the positive effect. Furthermore, there was a difference in the amount of flexural reinforcement; four groups were designed so that with a larger amount of flexural reinforcement, the sample could not be damaged by the rupture of the flexural reinforcement, and the importance and influence of the cross-wound CFRP were highlighted. It is also evident from the specimen dimensions that the potential of these larger diameter BFRP bars in thin HPC skin will not be fully utilized due to the small layer of HPC around the BFRP bar. These specimens exceed the reinforcement ratio limit for traditionally reinforced beams defined in Eurocode 2 [40]. Laboratory samples of short hollow beams 100 × 100 × 400 mm were loaded using a three-point bending test with a distance between supports of 300 mm.

## 2. Materials and Methods

### 2.1. Materials

#### 2.1.1. Concrete Mixture

By using a fine-grained HPC mixture with a maximal grain size of 1.2 mm, good penetration of the concrete matrix through the composite shear reinforcement mesh was ensured. The concrete mixture, as described in Table 1, achieved a compressive strength of 115.6 ± 4.3 MPa on cubes with an edge length of 100 mm, in accordance with the technical standard EN 12390-3:2019 [41]. The tensile strength, measured on prisms of 40 × 40 × 160 mm^3^ using three-point bending with a 100 mm support distance as per EN 12390-5:2019 [42], was 14.3 ± 0.9 MPa. The secant modulus of elasticity, measured on prisms of 100 × 100 × 400 mm^3^ in compression according to EN 12390-13:2021 [43], was 45.3 ± 0.7 GPa. All tests were conducted at 28 days of age using the Controls MCC-Multitest testing machine (Milan, Italy).

#### 2.1.2. Reinforcement

BFRP reinforcement bars as a bending reinforcement were used by the company ORLITECH^®^, produced by Binevir ITS Kompozit Üretim A.S. (Maslak, Turkey), and were used as tensile reinforcement for all specimens. The BFRP bars are made from thermosetting epoxy resin, with their surface modified using silica sand for higher diameters or wrapped from the yarn of the same material in the case of weaker reinforcement to enhance almost perfect bonding with the concrete matrix. The density of the BFRP bars is 2.0 g/mm^3^, with the basalt fiber content exceeding 80% by weight. Bars with diameters of 4 mm (treated using wave roving) and 8 mm (treated using silica sand) were used in this presented study. The 4 mm diameter BFRP bars have an average tensile strength of 1030 MPa, measured according to ISO 10406-1 [44] (the minimum declared tensile strength is 1000 MPa). The static modulus of elasticity, measured according to the same standard, is 55.8 GPa (with a minimum declared value of 50 GPa). The minimum declared bond strength with concrete is 25 N/mm^2^. The 8 mm diameter BFRP bars have an average tensile strength of 1265 MPa (minimum 1200 MPa). The static modulus of elasticity is 55.7 GPa (minimum 50 GPa), and the minimum declared bond strength with concrete is 50 N/mm^2^.

The cross-wound part of the composite reinforcement was prepared from carbon filament yarn with two different nominal linear density values of 1600 tex and 3700 tex. Carbon filament yarns Tenax™-E STS40 F13 24K 1600 tex were produced by Teijin Carbon Europe GmbH (Heinsberg, Germany), and ZOLTEK™ PX35 50K 3700 tex were produced by ZOLTEK Corporation (Bridgeton, NJ, USA). The declared tensile strength is 4300 MPa, and the modulus of elasticity is 240 GPa for the yarn with a lower nominal linear density [45]. These parameters for the higher tex yarn are 4137 MPa and 242 GPa [46]. The carbon yarn was impregnated using a two-component epoxy resin, SikaFloor-150^®,^ from the company Sika Deutschland GmbH (Stuttgart, Germany) to ensure proper interaction of all filaments. The resin has a flexural strength of 15 MPa and a modulus of elasticity of 2 GPa [47]. In previous research, a tensile test of CFRP from single roving was performed. Single carbon yarns were laminated by epoxy resin. Specimens were then placed into the steel sleeve for necessary anchoring in a testing machine. Specimens were placed into the testing machine, and in the middle of the roving was placed a potentiometer for exact determination of elongation. The measured and calculated tensile strength of CFRP from single roving was 3423 MPa, and the modulus of elasticity was 267 GPa [48].

### 2.2. Specimen Preparation

**Figure 1:**
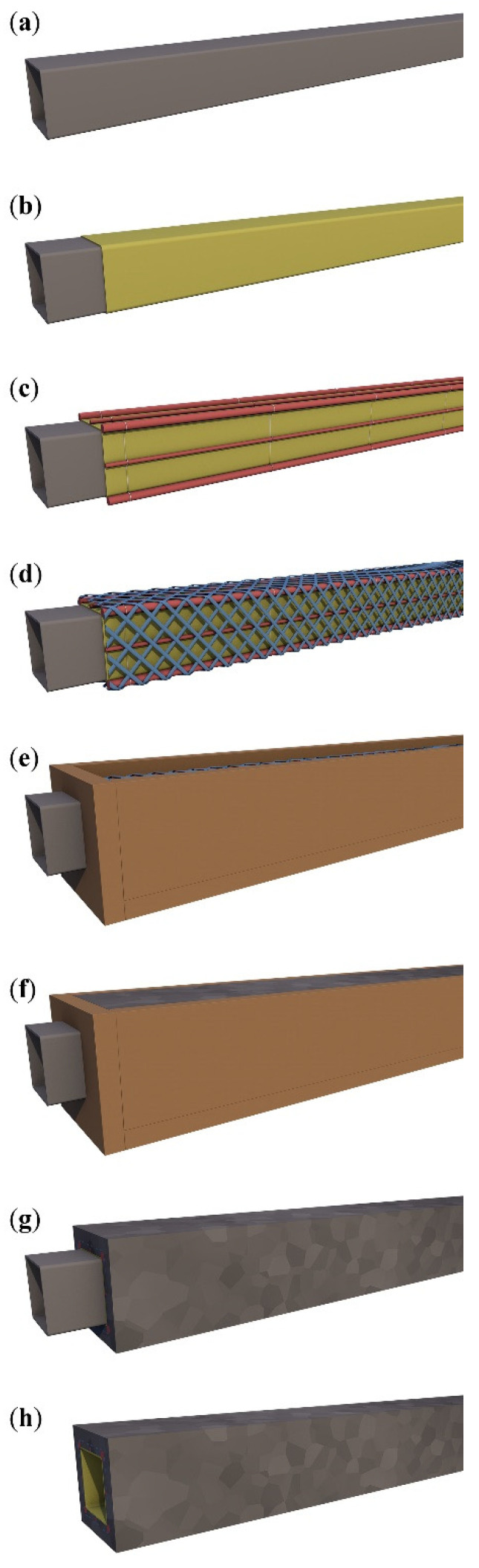
Specimen production process: (**a**) rigid steel core 60 × 60 mm^2^; (**b**) foam sheet separation skin wrapped on the steel core; (**c**) BFRP bending bars fixed by wire; (**d**) CFRP shear reinforcement and their homogenization; (**e**) installation of reinforcement into the mold; (**f**) concreting process; (**g**) demolding; (**h**) removal of the steel core.

The hollow beams were designed with a cross-section of 100 × 100 mm^2^ and a hollow core of 62 × 62 mm^2^. The cross-sectional laboratory dimension was inspired by previous research [49], the EN 12390-5:2019 standard [42], and also technological possibilities for the implementation of laboratory samples. While the weight is reduced to 61.7% compared to a solid element with similar outer dimensions, the elastic section modulus is only 14.8% lower, and the effective height can remain unchanged. The beams were manufactured at a length of 1200 mm and were then divided into specimens for testing purposes. Steel tubes 60 × 60 mm^2^ and a thickness of skin 2 mm were used as a rigid core for reinforcement preparation (Figure 1a). The steel tubes were wrapped with polyethylene foam sheets with a thickness of 1 mm, which resulted in a hollow core of 62 × 62 mm^2^ (Figure 1b) to provide separation of the steel core from the HPC matrix and allow easy removal. Then, bending BFRP reinforcement was fixed on the core using a thin steel wire (Figure 1c). The next step was the application of carbon woven reinforcement and homogenization using epoxy resin (Figure 1d) and the installation of the prepared wooden mold (Figure 1e). The next step was the casting of the HPC mixture (Figure 1f) and the next day after the concrete demolding (Figure 1g) and removal (pulling out) of the steel core (Figure 1h).

#### 2.2.1. Hollow Core

The beams’ hollow cores were formed using square steel tubes with dimensions of 60 × 60 × 2 mm^3^ as removable forms. The tubes were to be removed after concrete hardening and were covered with foam sheets with a thickness of 1 mm to prevent issues with their removal due to shrinkage of the concrete matrix. The steel tubes with separation layers were used as a platform for the tensile and shear reinforcement positioning.

#### 2.2.2. BFRP Tensile Reinforcement

**Figure 2:**
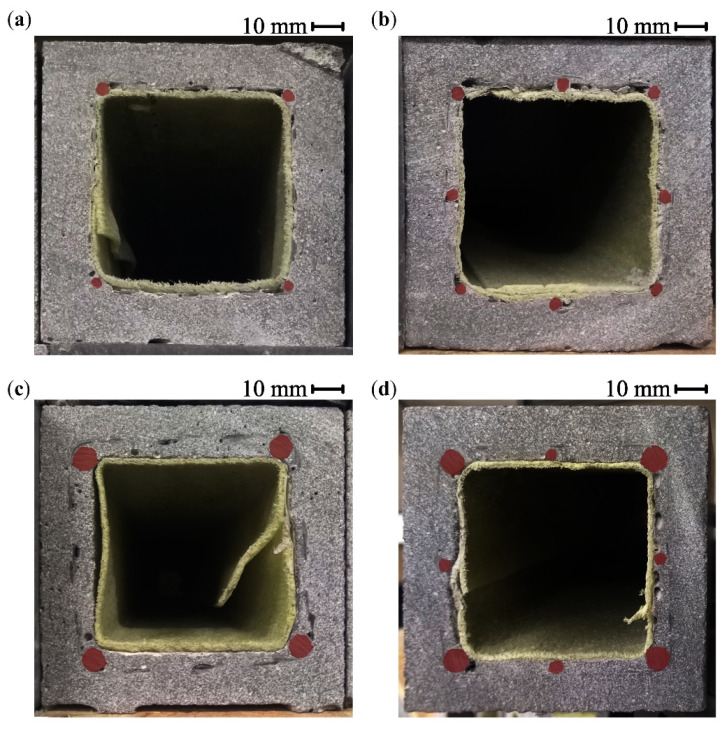
Hollow beam specimens with cross-wound CFRP shear reinforcement cross-sections: (**a**) 4Ø4 BFRP bars tensile reinforcement configuration; (**b**) 8Ø4 BFRP bars; (**c**) 4Ø8 BFRP bars; (**d**) 4Ø8 + 4Ø4 BFRP bars.

The BFRP bars were placed directly on the separation layer, and their position was secured using binding wire to prevent their shifting during the winding of the CFRP shear reinforcement. The BFRP reinforcement was prepared using bars with diameters of 4 and 8 mm in four variants shown in Figure 2 with different reinforcement ratios from 0.8% to 4.1% (regarding the hollow cross-section). Figure 2 also shows the position of the CFRP shear reinforcement for each variant of the tensile reinforcement.

#### 2.2.3. CFRP Shear Reinforcement

The carbon yarn was wound around the BFRP bars at an angle of ±45° from the longitudinal axis with 12 rovings in each direction. The rovings were impregnated with epoxy resin using a foam roller. The application of the foam roller limited accessibility to one side of the cross-wound carbon rovings. Before the epoxy resin was cured, it was coated from all directions with fine-grained silica sand with grain size from 0.1 to 0.6 mm to improve the interaction between the CFRP reinforcement and the concrete matrix.

**Figure 3:**
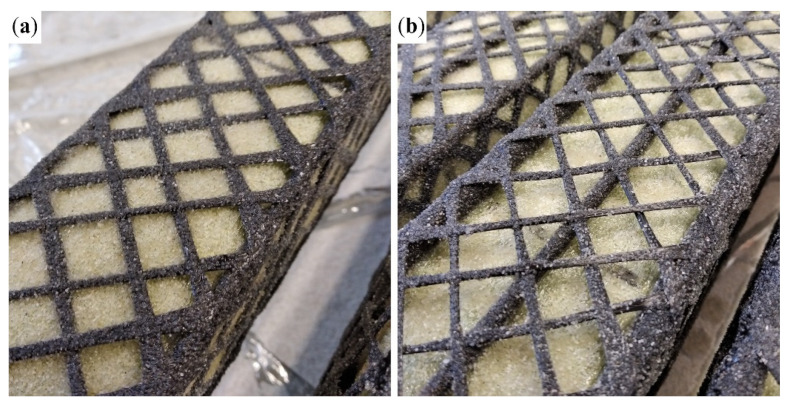
Sand-coated cross-wound 1600 tex CFRP reinforcement: (**a**) 4Ø4 BFRP bars configuration of tensile reinforcement with the CFRP reinforcement in contact with the separation layer of the temporary steel core; (**b**) 4Ø8 + 4Ø4 BFRP bars confirmation, where the CFRP reinforcement is positioned at a distance from the separation layer.

The Ø4 mm BFRP bars were pressed into the soft separation foam sheet during the winding of the shear reinforcement, which, for the 4Ø4 BFRP bars configuration, resulted in the impregnated carbon rovings being in direct contact with the separation layer, as shown in Figure 3a. The shear reinforcement will be, in this case, placed on the inner surface of the HPC shell, which will impact their interaction. The Ø8 mm BFRP bars provided better support and positioned the CFRP reinforcement at a distance from the separation layer (Figure 3b). This position of the shear reinforcement will allow for the concrete to fill the space between the BFRP bars and completely encase the CFRP reinforcement.

#### 2.2.4. HPC Shell

The temporary hollow core, mounted with tensile and shear reinforcement, was placed horizontally inside a mold, protruding through a faceplate on each side to secure its position. The concrete mixture was cast and vibrated to minimize the number of pores. The faceplates were removed, and the temporary steel core was pulled out of the hollow beam after 24 h. After removal from the mold, the beams were cut into specimens with a length of 400 mm.

**Figure 4:**
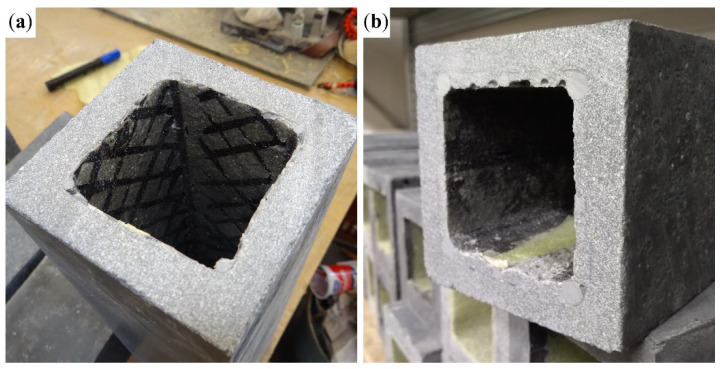
(**a**) 4Ø4 variant with exposed BFRP bars and cross-wound 1600 tex CFRP shear reinforcement; (**b**) 4Ø8 + 4Ø4 BFRP bars with cavities between the BFRP bars below the hollow core.

Figure 4a shows the variant with four BFRP bars with a diameter of 4 mm (Figure 2a) exposed to both CFRP shear reinforcement and BFRP tensile reinforcement as a result of their contact with the separation layer (Figure 3a). In the case of the 8Ø4 configuration, the middle BFRP bars raised the cross-wound CFRP reinforcement above the separation layer. As a result, the composite reinforcement was encased in the concrete matrix. Figure 4b shows the variant with 4Ø8 + 4Ø4 BFRP bars (Figure 2d), with cavities in the concrete matrix, where concrete could not fill the space between the BFRP bars below the hollow core. Variant 4Ø8 was similarly impacted.

### 2.3. Testing Methods

To evaluate the performance of the cross-wound CFRP shear reinforcement, all specimens were subjected to a 3-point bending test with a 300 mm distance between support pins. The experimental loading test was inspired by EN 12390-5:2019 [42] for determining the material properties of concrete. The small distance between the load supports in comparison with the larger beam height respects the focus of the experiment on the shear behavior of the specimen. The testing was controlled by an increment of displacement of 2 mm/min and was performed using the LabTest 4.100SP1 testing machine (LaborTech Ltd., Opava, Czech Republic). Table 2 provides an overview of all reinforcement configurations and a number of prepared specimens.

## 3. Results and Discussion

**Figure 5:**
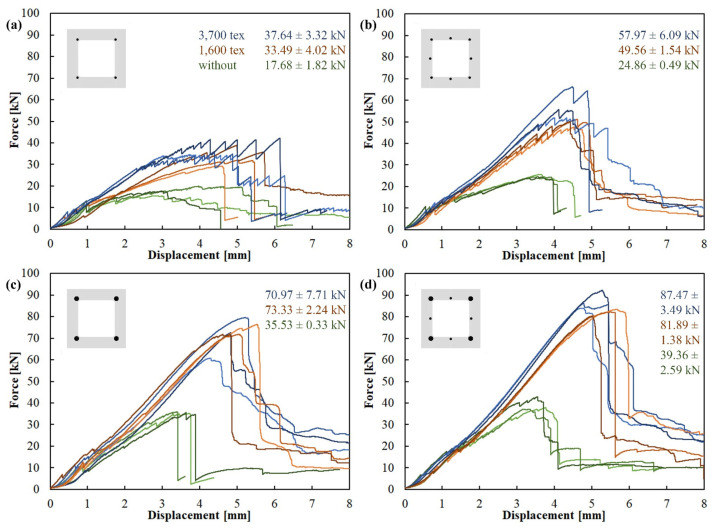
Force/displacement diagram of the 3-point bending test with a support pin span of 300 mm: (**a**) 4Ø4 BFRP bars tensile reinforcement configuration; (**b**) 8Ø4 BFRP bars; (**c**) 4Ø8 BFRP bars; (**d**) 4Ø8 + 4Ø4 BFRP bars. Color indicates shear reinforcement—without (green), 1600 tex (red), 3700 tex (blue).

The three-point bending test resulted in all specimens without shear reinforcement in a shear failure. The development of oblique shear cracks always leads to the formation of a shear crack along the tensile reinforcement and its subsequent pullout. For specimens with shear reinforcement, specimens with the lowest tensile reinforcement ratio failed in tension, while all other tensile reinforcement configurations failed in shear. The mode of failure and ultimate bending strength of all specimens are summarized in Table 2 and force/displacement diagrams in Figure 5.

**Figure 6:**
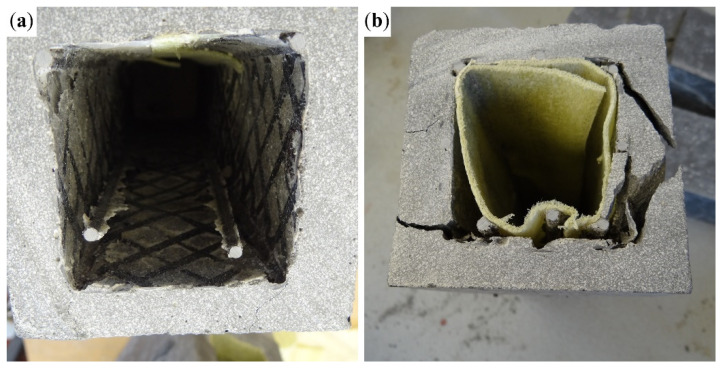
(**a**) Delamination of the tensioned BFRP bars inside the hollow core of the 4Ø4–16 configuration; (**b**) shear cracks along the length of the BFRP bars and their pullout from the concrete matrix in the 8Ø4–16 configuration.

In the specimens with only 4Ø4 BFRP tensile reinforcement bars (Figure 5a), tensile failure occurred in both variants of the shear reinforcement as a result of delamination of the tensile bars, as shown in Figure 6a. This happened due to the small contact area of the BFRP bars with the surrounding concrete matrix. In the samples with 8Ø4 bars (Figure 5b), simple delamination of the BFRP bars no longer occurred. The reason was a better interaction of the middle bar of the tensile reinforcement with the concrete matrix due to a larger contact area. In these specimens, tensile reinforcement lost cohesion with the concrete matrix as a result of the development of a shear crack along the reinforcement (Figure 6b).

**Figure 7:**
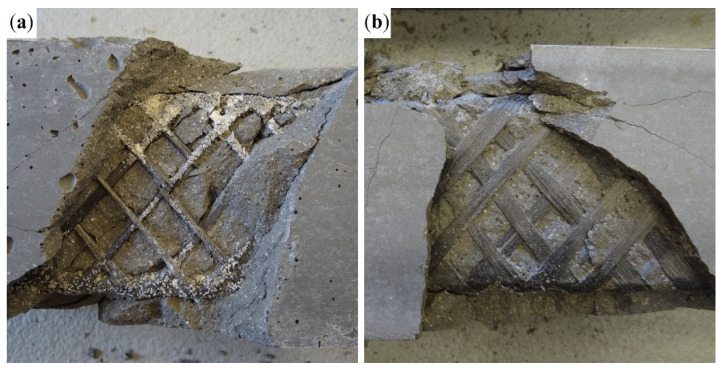
Comparison of shear failure of specimens with 4Ø8 BFRP tensile reinforcement: (**a**) Cross-wound 1600 tex CFRP shear reinforcement with ruptured carbon rovings transverse to the shear crack; (**b**) 3700 tex CFRP shear reinforcement with missing sand coating as a result of delamination of the concrete matrix.

The shear reinforcement was fully utilized in the case of specimens with tensile reinforcement configuration 8Ø4 (Figure 5c) and 4Ø8 + 4Ø4 (Figure 5d). Specimens with both 1600 tex and 3700 tex behaved similarly and achieved comparable results, as shown in Figure 5c,d. All of them collapsed due to a shear failure. The specimens with 1600 tex shear reinforcement failed as predicted, with the rupture of carbon rovings transverse to the shear cracks (Figure 7a). The specimens with 3700 tex shear reinforcement failed due to delamination of the concrete matrix from the shear reinforcement, while the shear reinforcement remained undamaged. Figure 7b shows that the concrete matrix delaminated alongside the sand coating of the CFRP reinforcement.

## 4. Conclusions

The hollow HPC beams with 38% lower weight compared to solid beams with the same outer dimensions can retain the same effective height while experiencing slightly sooner crack development in the concrete matrix due to a 14.8% elastic section modulus. The main drawback of the hollow cross-section is the reduced shear capacity, as the thickness of the HPC shells in the middle of the cross-section totals only 38% of the solid element. The cross-wound CFRP shear reinforcement in both variants significantly increased the shear capacity of the hollow beams. The temporary core, which was used to form the beams’ hollow core, also provided an efficient way of reinforcement positioning, as the tensile and shear reinforcements were mounted directly on the temporary core. The reinforcement was securely positioned without any additional spacers needed to maintain the designed distance from the mold surface.

- The position of the tensile reinforcement in contact with the surface of the hollow core proved to be problematic, as it increased the risk of pullout of the BFRP bars. This issue was most pronounced in the case of the Ø4 mm BFRP bars, which were pressed into the soft separation layer of the hollow core during positioning, which resulted in a larger exposed surface area and, therefore, a smaller contact area with the concrete matrix.
  1. The process of epoxy resin impregnation of the carbon rovings using a foam roller, which could reach the cross-wound textile reinforcement only from one side, resulted in the case of the 3700 tex rovings in insufficient penetration of the yarn. The surface of the impregnated rovings did not contain enough resin to properly bond with the silica sand meant to coat it. This led to the delamination of the concrete matrix along with the silica sand, yielding results only slightly higher compared to specimens with 1600 tex reinforcement, meaning that the shear capacity of the 3700 tex reinforcement was not fully utilized.
  2. The cross-wound CFRP shear reinforcement made of 1600 tex carbon rovings was, in the case of specimens with a 1.6% to 4.1% tensile reinforcement ratio, fully utilized, as all of these specimens failed due to a rupture of the carbon rovings transverse to the shear crack. These specimens had 100% or higher ultimate mean flexural strength compared to specimens without any shear reinforcement.
- The spalling of concrete fragments as a result of stress release during the rupture of the shear reinforcement also showed that the 1600 tex rovings were sufficiently impregnated with the epoxy resin, as the sand coating did not delaminate along with the concrete matrix, as was the case for specimens with the 3700 tex CFRP reinforcement.

The position of the tensile reinforcement directly on the surface of the temporary core led to several issues, especially in the case of the bars with smaller diameters. It would be advantageous to position both the tensile and shear reinforcement closer to the outer surface of the hollow beam. This would lead to a higher effective height of the element and, therefore, higher flexural capacity, while the position further from the surface of the HPC shell would provide better interaction with the shear reinforcement. Other means of epoxy resin application should also be explored to ensure proper impregnation of yarn with a higher number of filaments.

## Figures and Tables

**Table 1:** High-performance concrete mix composition.

Component	[kg/m^3^]
Cement I 42.5 R	752
Technical silica sand	1062
Silica flour	250
Silica fume	110
Superplasticizers	29
Water	176

**Table 2:** Overview of all prepared specimens.

Diagram	Tensile Reinforcement	Cross-Sectional Area Reinforcement Ratio	Shear Reinforcement	Amt.	Ultimate Force [kN]	Failure
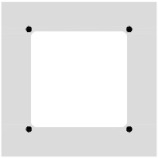	**4Ø4** BFRP bars	50.26 mm^2^ A_R_/A_C_ = 0.81%	**00**–without	3	17.68 ± 1.82	Shear
			**16**–1600 tex	3	33.49 ± 4.02	Tensile
			**37**–3700 tex	3	37.64 ± 3.32	Tensile
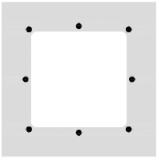	**8Ø4** BFRP bars	100.53 mm^2^ AR/AC = 1.63%	**00**–without	3	24.86 ± 0.49	Shear
			**16**–1600 tex	3	49.56 ± 1.54	Shear
			**37**–3700 tex	3	57.97 ± 6.09	Shear
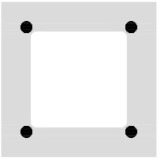	**4Ø8** BFRP bars	201.06 mm^2^ AR/AC = 3.26%	**00**–without	3	35.53 ± 0.33	Shear
			**16**–1600 tex	3	73.33 ± 2.24	Shear
			**37**–3700 tex	3	70.97 ± 7.71	Shear
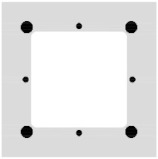	**4Ø8 + 4Ø4** BFRP bars	251.33 mm^2^ AR/AC = 4.08%	**00**–without	3	39.36 ± 2.59	Shear
			**16**–1600 tex	3	81.89 ± 1.38	Shear
			**37**–3700 tex	3	87.47 ± 3.49	Shear

## Data Availability

The original contributions presented in the study are included in the article, further inquiries can be directed to the corresponding author.
